# Demographic monitoring of wild muriqui populations: Criteria for defining priority areas and monitoring intensity

**DOI:** 10.1371/journal.pone.0188922

**Published:** 2017-12-13

**Authors:** Karen B. Strier, Carla B. Possamai, Fernanda P. Tabacow, Alcides Pissinatti, Andre M. Lanna, Fabiano Rodrigues de Melo, Leandro Moreira, Maurício Talebi, Paula Breves, Sérgio L. Mendes, Leandro Jerusalinsky

**Affiliations:** 1 Department of Anthropology, University of Wisconsin-Madison, Madison, Wisconsin, United States of America; 2 Departamento de Ciências Biológicas, Universidade Federal de Espírito Santo, Vitória, Espírito Santo, Brazil; 3 Preserve Muriqui, Caratinga, Minas Gerais, Brazil; 4 Muriqui Instituto de Biodiversidade, Caratinga, Minas Gerais, Brazil; 5 Centro de Primatologia de Rio de Janeiro (CPRJ–INEA), Guapimirim, Rio de Janeiro, Brazil; 6 Programa de Pós-Graduação em Ecologia, Universidade Federal do Rio de Janeiro, Rio de Janeiro, Brazil; 7 Instituto de BioCiências, Universidade Federal de Goias, Regional Jataí, Jataí, Goias, Brazil; 8 Universidade Federal de São Paulo, Campus Diadema, Laboratório de Primatologia e Conservação de Espécies, Departamento de Ciências Ambientais & Programa de Pós-Graduação Análise Ambiental Integrada, Diadema São Paulo, Brazil; 9 Instituto Pró-Muriqui, São Miguel Arcanjo, São Paulo, Brazil; 10 Ecoatlântica, Rio de Janeiro, Rio de Janeiro Brazil; 11 Instituto de Pesquisas da Mata Atlântica–IPEMA, Vitória, Espírito Santo, Brazil; 12 Centro Nacional de Pesquisa e Conservação de Primatas Brasileiros–CPB, Instituto Chico Mendes de Conservação da Biodiversidade–ICMBio, João Pessoa, Paraíba, Brazil; University of Massachusetts Amherst, UNITED STATES

## Abstract

Demographic data are essential to assessments of the status of endangered species. However, establishing an integrated monitoring program to obtain useful data on contemporary and future population trends requires both the identification of priority areas and populations and realistic evaluations of the kinds of data that can be obtained under different monitoring regimes. We analyzed all known populations of a critically endangered primate, the muriqui (genus: *Brachyteles*) using population size, genetic uniqueness, geographic importance (including potential importance in corridor programs) and implementability scores to define monitoring priorities. Our analyses revealed nine priority populations for the northern muriqui (*B*. *hypoxanthus*) and nine for the southern muriqui (*B*. *arachnoides*). In addition, we employed knowledge of muriqui developmental and life history characteristics to define the minimum monitoring intensity needed to evaluate demographic trends along a continuum ranging from simple descriptive changes in population size to predictions of population changes derived from individual based life histories. Our study, stimulated by the Brazilian government’s National Action Plan for the Conservation of Muriquis, is fundamental to meeting the conservation goals for this genus, and also provides a model for defining priorities and methods for the implementation of integrated demographic monitoring programs for other endangered and critically endangered species of primates.

## Introduction

Accurate assessments of the conservation status of primate taxa depend on data about changes in the sizes and fragmentation of populations [1]. These assessments rely on the documentation of trends, such as declining population numbers and increasing population fragmentation, for estimating a taxon’s risks of extinction. Additional information, such as data on changes in sex ratios or in the proportion of reproductive (versus non-reproductive) females, can be critical for predicting the probability of future population growth or decline. Even more detailed data on age-specific or individual-specific patterns of mortality and fertility can contribute to more accurate projections about a population’s potential for persistence over time [2].

Obtaining the data needed to evaluate these demographic trends and their implications for the conservation of endangered species requires standardized methods for the systematic monitoring of populations living under different conditions. Ideally, all populations of endangered and critically endangered species would be monitored closely enough to detect the first signs of real or projected risks, thereby facilitating rapid implementation of tactics aimed at mitigating the causes of a population’s anticipated decline. In reality, however, limited resources may make it impossible to monitor and respond to risks for more than a subset of the remaining populations. Identifying which populations have the greatest probability of ensuring a species’ persistence and therefore merit the closest monitoring is thus a necessary step in any species’ conservation action plan.

The importance of obtaining reliable demographic data was recognized in the Brazilian National Action Plan for the Conservation of Muriquis (*Plano de Ação Nacional para a Conservacao dos Muriquis*, PAN Muriquis) [3], which focuses on the protection of the two species of muriquis (*Brachyteles hypoxanthus* and *B*. *arachnoides*), both of which are endemic to the Atlantic forest and classified as critically endangered primates [4,5]. Specific actions of the PAN Muriquis [3] call for the identification of priority areas for demographic monitoring (Action 5.1), and for the development of methods for an integrated demographic monitoring program (Action 5.2). Here, we describe our criteria for the definition of priority areas and our rationale and protocols for optimal monitoring intensities, using published and first-hand knowledge of each site. Although focused on muriquis, our rationales, criteria, and monitoring approaches are widely applicable for many other species. We hope our approach will facilitate similar efforts for prioritizing and implementing the demographic monitoring of other endangered primate species.

## Materials and methods

**Fig 1:**
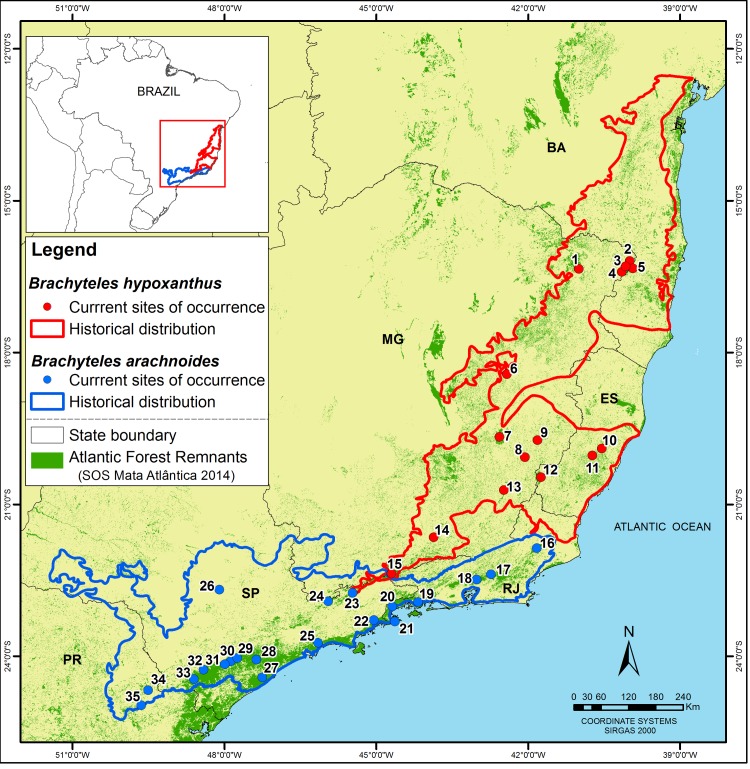
Localities of known muriqui populations. See Table 1 for names and coordinates of each site. Historical extent of occurrence is from [6].

To define priority areas for the demographic monitoring of all known muriqui populations (Fig 1, Table 1), we expand on the demographic and geographic criteria used by the IUCN Standards and Petitions Subcommittee [1] in their evaluations of the threatened status of taxa. Specifically, our assessments of priority areas for monitoring are based on the following criteria: (i) Population size (and composition); (ii) Genetic uniqueness of the population; and (iii) Geographic importance of the population (ecological uniqueness of the habitat and/or its strategic location for optimizing connectivity).

**Table 1:** Locality names and coordinates of known populations of the northern muriqui (*Brachyteles hypoxanthus*) and southern muriqui (*B*. *arachnoides*). Points correspond to locations in Fig 1.

POINT	LOCALITY	STATE	LATITUDE	LONGITUDE	AREA (ha)	POPULATION (N mature individuals)
1	REBIO MATA ESCURA	MG	-16.3500	-41.0000	51,000	<50*
2	PARQUE ESTADUAL ALTO CARIRI	MG	-16.3166	-39.9951	6,100	<50*
3	RPPN FAZENDA LOREDANO ALEIXO	MG	-16.4200	-40.0507	575	<50*
4	REVIS MURIQUIS	MG	-16.4300	-40.0791	2,722	<50*
5	PARNA ALTO CARIRI	BA	-16.3333	-39.9833	19,220	<50*
6	PEÇANHA	MG	-18.4295	-42.4205	420	<50*
7	PARQUE ESTADUAL DO RIO DOCE	MG	-19.6667	-42.5667	36,970	<100*
8	RPPN MATA DO SOSSEGO	MG	-20.0700	-42.0812	180	<50*
9	RPPN FELICIANO MIGUEL ABDALA	MG	-19.7333	-41.8167	957	<250*
10	REBIO AUGUSTO RUSCHI	ES	-19.9000	-40.5500	4,700	<50
11	ÁREAS PARTICULARES EM SANTA MARIA DE JETIBÁ	ES	-20.0333	-40.7333	1,000	<50*
12	PARNA CAPARAÓ	MG	-20.4667	-41.7500	32,000	<100*
13	PARQUE ESTADUAL DA SERRA DO BRIGADEIRO	MG	-20.7167	-42.4833	15,000	<100*
14	RESERVA DO IBITIPOCA	MG	-21.6500	-43.8700	32	<50
15	PARNA ITATIAIA	MG, RJ	-22.3667	-44.7000	28,086	<50
16	PE DO DESENGANO	RJ	-21.8667	-41.8333	22,400	<50
17	PE TRÊS PICOS, RESERVA ECOLÓGICA GUAPIAÇU	RJ	-22.3833	-42.7333	46,850	<50
18	PARNA SERRA DOS ÓRGÃOS	RJ	-22.4833	-43.0167	20,020	<100*
19	PE CUNHAMBEBE	RJ	-22.9276	-44.1761	38,054	<50
20	PARNA SERRA DA BOCAINA	RJ, SP	-23.0167	-44.6833	104,045	<50
21	APA DO CAIURUÇU, RESERVA ECOLÓGICA DA JUATINGA	RJ	-23.3167	-44.6333	42,552	<50
22	PE SERRA DO MAR	SP	-23.2833	-45.0500	315,391	<100
23	FAZENDA SÃO SEBASTIÃO DO RIO GRANDE	SP	-22.7500	-45.4667	1,206	<50*
24	APA MUNICIPAL SÃO FRANCISCO XAVIER	SP	-22.9167	-45.9500	10,000	<50
25	PARQUE DAS NEBLINAS	SP	-23.7333	-46.1500	2,100	<50*
26	FAZENDA BARREIRO RICO	SP	-22.6833	-48.1000	2,325	<50*
27	EE JURÉIA-ITATINS	SP	-24.4167	-47.2500	79,240	<50
28	LEGADO DAS ÁGUAS VOTORANTIM RESERVE	SP	-24.0685	-47.3650	31,000	<100*
29	FAZENDA SÃO MIGUEL	SP	-24.0321	-47.9022	2,700	<50*
30	PE CARLOS BOTELHO	SP	-24.1314	-47.9492	37,644	<250*
31	ECOPARQUE MURIQUI	SP	-24.0922	-47.9743	100	<50*
32	PE INTERVALES	SP	-24.2684	-48.4138	42,988	<50
33	PE TURÍSTICO DO ALTO RIBEIRA	SP	-24.4500	-48.6000	34,800	<50
34	FAZENDA OLHO D'ÁGUA	PR	-24.6703	-49.5044	700	<50
35	FAZENDA JOÃO PAULO II	PR	-24.9685	-49.6418	2,908	<50

Abbreviations for conservation units: PE = Parque Estadudal; REVIS = Refúgio de Vida Silvestre; RPPN = Reserva Particular Patrimonio Natural; PARNA = Parque Nacional; REBIO = Reserva Biologica; EE = Estação Ecologica. Abbreviations for states: MG = Minas Gerais; BA = Bahia; ES = Espirito Santo; RJ = Rio de Janeiro; SP = São Paulo; PR = Paraná. Areas are updated from [3]. Population data are distinguished based on authors’ data (*) or estimates, following categories used by IUCN [1]. See also Table 2 and Table 3.

In addition, for each population that meets at least one of the priority criteria, we assessed the (iv) feasibility of implementing a systematic demographic monitoring program. This assessment of “implementability” is based on current knowledge of accessibility and logistics and thus provides a basis for identifying sites where demographic monitoring would be most likely to succeed.

The initial demographic data collected in any population will correspond to the first count of individuals in the population [7]. However, even among areas identified as priorities, the frequency of monitoring and the degree to which individuals can be recognized will affect the kinds of questions that can be addressed. To identify criteria for determining the optimal monitoring intensity, we drew on our collective experiences of observing wild northern and southern muriquis and considered (i) the degree to which individuals in the population can be identified; (ii) the frequency of monitoring; and (iii) the period of time over which the population is monitored. We then used these considerations to evaluate the information that each level of monitoring intensity can yield.

### Criteria for defining priority areas

#### Population size (and composition)

The IUCN Standards and Petitions Subcommittee [1] defines a taxon’s population size as “the number of mature individuals.” However, we distinguish (by sex) other age classes in our demographic assessments because information on the composition of a population can provide insights into its potential for growth versus decline (see [2] for evidence from the RPPN-FMA population of muriquis).

The IUCN Red List criteria consider the smallest populations (<50 mature individuals) and populations that are declining in size to be at the greatest risks of extinction. Therefore, we prioritize the largest known populations (≥ 100 individuals, ≥50 mature individuals), which should have the greatest potential for persistence (assuming all other conditions across populations are equal) for systematic demographic monitoring of trends in population size (e.g., increasing or decreasing) or in composition (e.g., shift in sex ratios or in proportion of immature versus mature individuals).

#### Genetic uniqueness

Only limited data on population genetics are available for either species of muriqui. Nonetheless, comparative analyses permit us to identify some initial priority populations based on the uniqueness and diversity of haplotypes across a subset of populations of each species. Analyses of the mitochondrial DNA control region from 152 northern muriqui individuals from eight populations revealed that the total number of haplotypes (3–8, of 23 haplotypes), the number of unique haplotypes (3–5), and haplotype diversity (0.626–0.846) were highest in the populations occupying the largest forests. However, 42% of the individuals sampled were from one population (RPPN Feliciano Miguel Abdala), which could influence these results [8].

Preliminary genetic analyses of 60 southern muriqui individuals from 10 populations found 39 haplotypes in 612bp of the mtDNA Control Region, with haplotype diversity of 0.976 and 13 out of 14 nuclear microsatellite loci polymorphic in 55 individuals, an average of 6.86 alleles (range = 3–12 alleles) and observed heterozygosity ranging from 0.074 to 0.778 [9]. These results indicate a high diversity with no clear evidence of geographical structuring and one population (PE Carlos Botelho) as possessing most of this diversity, with no concrete evidence of a recent genetic bottleneck for this population inhabiting the largest forest for this species [9]. Thus, in contrast to the very patchy distribution of genetic diversity of the northern muriqui, there is still at least one population of the southern muriqui in which most of this species’ genetic diversity is represented. However, almost 60% of the sampled individuals were obtained from this population, which could bias the results.

Further genetic studies are clearly needed to better understand both the historical and current genetic relationships among extant populations of each species, and to resolve the relationships between the two species, including specifically whether both species occur in the state of Rio de Janeiro [6,10,11]. We anticipate that any populations of northern and southern muriquis identified as having occurred sympatrically in the past (with the potential for hybridization) would also fall within our criteria for monitoring priority. Reasons such as these resulted in our scoring the genetic uniqueness of a population for which no genetic data are yet available as “expected” for both species.

#### Geographic importance

We recognize two additional features of extant populations that may be of critical importance to the long-term conservation of muriquis, and therefore lead us to include these populations in our priorities for long-term monitoring. First, populations at the extreme of the species distribution may be most vulnerable to extinction because they are susceptible to the most extreme ecological conditions (e.g., natural or anthropogenic pressures) at present. We define extreme populations as those that occur at the most extreme latitudinal, longitudinal, and altitudinal ranges of each species.

The projected effects of climate change (e.g., increasing temperatures and drought) on local vegetation may also make some of these extreme populations critical to the long-term survival of muriquis. This is because central areas in the species’ distributions today may be less suitable habitats, while areas at the geographic extremes may become more optimal habitats as temperature, rainfall, and seasonality patterns begin to shift [6,12]. The warming trend documented at one important northern muriqui locality (RPPN Feliciano Miguel Abdala) illustrates the potential for suitable habitats to shift [13]. Other extant populations may be even more important to include immediately in our monitoring priorities because of their key roles in optimizing current and anticipated forest corridor projects, such as Legado das Águas Votorantim Reserve for southern muriquis.

#### Implementability

For each population that meets the demographic, genetic, or ecological criteria, or is expected to meet one or more of these criteria as more information about the population becomes available, we also consider criteria that facilitate or represent obstacles to long-term monitoring. The implementability of demographic monitoring can be assessed qualitatively from current knowledge based on three parameters: 1) Whether researchers have access (e.g., in terms of trails, permission to enter the forest) to monitor the muriqui population at a site; 2) Whether there is institutional encouragement and support for the monitoring; and 3) Whether monitoring of the population is considered to be feasible in terms of terrain, logistics and personnel or financial resources. When all three of these parameters are met (i.e., Implementability = 1,2,3), demographic monitoring of the population is considered to be highly feasible; when no parameters are met (Implementability = 0), it may be difficult or impossible to implement the monitoring program without improving accessibility and logistical issues.

### Criteria for determining monitoring intensity

#### Level of individual identification

The specificity of demographic data obtained can range from counts of the number of animals observed, to counts of the number of animals represented in different age and/or sex classes, to counts of distinct individuals, whose age-sex classes may or may not be known to precision. The ability to distinguish individuals usually requires repeated observations over extended periods of time, whereas the ability to identify animals by age and sex class may be possible based on visible physical characteristics that correspond to obvious developmental stages. Sometimes these physical traits can be combined with landmarks in behavioral development, documented for that species, such as the age at which infants shift from being carried ventrally to dorsally. Examples of these behavioral and physical characteristics for distinguishing muriqui age-sex classes are provided in the Supporting Information (S1 Table and Figures A-R in S1 File).

#### Monitoring frequency

The frequency of demographic monitoring is defined as the number of counts made per unit time. These may occur daily or nearly daily, in the case of continuous field studies, or at longer intervals, in the case of targeted expeditions. Counts of the same populations over time will permit analyses of demographic trends. The shorter the intervals between successive counts, the more precise the estimates of demographic trends will be. However, factors such as limited resources for maintaining the continuity of observations and poor implementability may necessitate less frequent monitoring.

#### Monitoring duration

Variation in monitoring duration, or the period of time over which the population is monitored, will affect the accuracy of detecting real demographic trends. We used the median interbirth interval to calibrate the minimum monitoring duration needed for assessing population trends, and the median ages of first birth for females and first reproduction (based on first complete copulation or paternity) for males to set the minimum monitoring duration to evaluate the role of individual life histories in demographic trends.

## Results and discussion

### Priority areas

#### Northern muriqui monitoring priorities

The largest populations of northern muriquis (≥ 100 individuals total; ≥ 50 mature individuals), and thus, those prioritized for systematic monitoring based on demographic criteria, are summarized in Table 2. The same four populations (RPPN-FMA, PESB, PERD, and SMJ) were also prioritized as genetically “discrete management units” in an analysis of genetic data from eight populations of northern muriquis [8]. Additional analyses of the genetics of other populations of northern muriquis may lead to adjustments or additions to this list.

**Table 2:** Priority populations for the demographic monitoring of the northern muriqui (Cells in bold are priorities).

Population	Size [see legend for count data]	Genetic uniqueness [N haplotypes (N hapl), N unique haplotypes(N unique), haplotype diversity (*h*), data from^8^]	Extreme habitat [altitude, latitude]	Corridor anchor	Implementability
Reserva Particular do Patrimônio Natural Feliciano Miguel Abdala, Caratinga, Minas Gerais (RPPN- FMA)^1^	**N = 335**	**N hapl = 3,** **N unique = 3,** ***h* = 0.626**	<1,000m.a.s.l., -19.7333	**With RPPN-MS**	1,2,3
Parque Estadual da Serra do Brigadeiro, Minas Gerais (PESB)^2^	**N = 325**	**N hapl = 7,** **N unique = 5,** ***h* = 0.846**	<2,000m.a.s.l., -20.7167	None at present	1,2,3
Parque Estadual do Rio Doce, Minas Gerais (PERD)^3^	**N = 132**	**N hapl = 8,** **N unique = 5,** ***h* = 0.818**	<1,000m.a.s.l., -19.6667	None at present	1,2
Santa Maria do Jetibá, Espirito Santo (SMJ)^4^	**N = 115**	**N hapl = 7,** **N unique = 5,** ***h* = 0.754**	<1,000m.a.s.l., -20.0333	**With REBIO Augusto Ruschi**	1,2,3
Parque Nacional do Caparaó, Minas Gerais and Espírito Santo^5^	**N≥82**	N hapl = 2, N unique = 0, *h* not calculated	**>2,000m.a.s.l.,** **(highest altitude)** -20.4667	None at present	1,2
Reserva Biológica Augusto Ruschi, Espírito Santo (REBIO Augusto Ruschi)	N<50 (estimate)	Not known	<1,000m.a.s.l., -19.9000	**With SMJ**	1,2,3
Reserva Particular do Patrimônio Natural Mata do Sossego, Minas Gerais (RPPN-MS)^6^	N = 42	N hapl = 1, N unique = 0, *h* = 0.000	<2,000m.a.s.l., -20.0700	**With RPPN FMA**	1,2,3
Reserva Biológica da Mata Escura, Minas Gerais (REBIO ME)^7^	N<50 (estimate)	Not known, but expected based on geographic extreme and isolation	<1,000m.a.s.l., **-16.3500 (largest forest among northernmost latitudes)**	None at present	1
Parque Nacional do Itatiaia, Minas Gerais and Rio de Janeiro	N<50 (estimate)	Not known	<2,000m.a.s.l., **-22.3667 (southernmost latitude)**	Possible corridor pending data on species	1,2,3

^1^RPPN-FMA: Based on complete count as of May 2013; data from K. B. Strier; updated from [14].

^2^PESB: Estimated by sweep and aerial census; data from Melo et al. [15].

^3^PERD: Estimated by sweep census; data from Dias, et al. [16]; updated in [17].

^4^SMJ: Counting in progress as of July 2013; data from S. L. Mendes; updated from Mendes et al. [18].

^5^PARNA Caparaó: Data from Mendes et al. [19]

^6^RPPN-MS: Estimated as of December 2012; data from Tabacow and Melo [20].

^7^REBIO ME: Estimated based on Melo [21].

Different populations emerge from considerations of geographic importance (Table 2). For example, Mata Escura in northeastern MG is the northernmost population of *Brachyteles hypoxanthus* and of the genus, and northern muriquis in Parque Nacional do Caparaó (up to 2,000 m above sea level) inhabit the highest altitudinal range. The most obvious population for its role in forest expansion and corridor projects is the RPPN-MS, which remains as an anchor for the Caratinga-Sossego corridor project. The forest of REBIO Augusto Ruschi might emerge as similarly important as an anchor for the metapopulation of muriquis in SMJ.

Assessment of the implementability of demographic monitoring at each site reveals high overlap with three of the populations we prioritized on the basis of demographic and genetic criteria (RPPN-FMA, PESB, SMJ). Nonetheless, there are barriers to monitoring at three sites including the most extreme altitude and northernmost latitude sites.

#### Southern muriqui monitoring priorities

The largest populations of southern muriquis are prioritized for systematic monitoring, although some are suspected but not yet known to meet the demographic criteria of ≥ 100 individuals total; ≥ 50 mature individuals (Table 3). In addition, new information suggests that there may be other areas that support larger populations than previously suspected (e.g., PE do Desengano, Tres Picos, PE Cunhambebe, PARNA Serra da Bocaina; see Fig 1 and Table 1).

**Table 3:** Priority populations for the demographic monitoring of the southern muriqui. (Cells in bold are priorities).

Population	Size [see legend for count data]	Genetic uniqueness [N haplotypes (N hapl), N unique haplotypes (N unique), haplotype diversity (*h*), data from^9^]	Extreme habitat [altitude, latitude, longitude]	Corridor anchor	Implementability
PE Carlos Botelho, SP (PECB)^1^	**N = 450**	**Highest diversity known, N hapl = 39,** **N unique = not known, *h* = 0.976**	Core population occurs at<800m.a.s.l., -24.1314, -47.9492	**With Fazenda São Miguel & EcoParque Muriqui**	1,2,3
Legado das Águas Votorantim Reserve, SP^2^	**N≤100**	Not known	<500m.a.s.l., -24.0685, -47.3650	None at present	1,2,3
EE Juréia-Itatins, SP	N<50 (estimate)	Not known	Sea level up to <300m.a.s.l., -22.6833, -47.2500	None at present	1,2
Fazenda Barreiro Rico, SP	N<50 (estimate)	Not known, but possibly low diversity and high genetic uniqueness based on geographic extreme and isolation	<600m.a.s.l., **-22.6833, -48.1000 (one of the westernmost longitudes)**	**Western Anchor corridor pending data on species**	0
Fazenda Fibria São Sebastião, SP^3^	N<50	**Not known, but expected to be very low diversity and high genetic uniqueness based on geographic extreme and historic isolation**	**>2,000m.a.s.l., (one of highest altitudes)** -22.7500, -45.4667	None at present	1,2,3
Parque das Neblinas, SP^4^	**N<50**	Not known	<300m.a.s.l. -23.7333, -46.1500	None at present	1,2,3
PE Serra do Mar, Caraguatatuba, SP	**N<100 (estimate)**	Possibly diverse, not known	Sea level to <200m.a.s.l., -23.2833, -45.0500	None at present	1,2,3
Castro, Paraná	N<50	Not known, but possibly low diversity and high genetic uniqueness based on geographic extreme and isolation	<1,000m.a.s.l., **-24.9685, -49.6418** **(southernmost latitude, westernmost longitude)**	**With PECB**	0
PN Serra dos Órgãos, RJ (PARNASO)^5^	**N<50 (estimate)**	**Possibly diverse; see legend**	**>2,000m.a.s.l. (one of highest altitudes)** -22.4833, -43.0167	None at present	1,2,3
PE Desengano, RJ	N<50 (estimate)	Not known	<500m.a.s.l. **-21.8667, -41.8333** **(northernmost latitude)**	**With PARNASO**	1,2,3

^1^PECB: Estimated by line transects and long-term analyses; data from Talebi & Lee [22].

^2^Legado das Águas Votorantim Reserve: Population estimated by initial study and demographic monitoring; see Talebi et al [23].

^3^ Fazenda Fibria São Sebastião: N = 47 individuals, approximately 23 of which are mature; see Talebi & Soares [24]; this site, is located in the Mantiqueira hills, which span the states of São Paulo and Minas Gerais, is potentially important in the context of historical distributions and past refugia [6, 25]

^4^Parque das Neblinas: Despite the relatively small population size, this is the only population in the Serra do Mar being monitored, see Talebi [26].

^5^Serra dos Órgãos: Prioritized because of the possibly that it is the largest remaining population in the state of Rio de Janeiro and therefore the most genetically diverse in the state; estimated by Breves & Pissinatti [27].

Preliminary genetic analyses of southern muriquis indicate that much of the genetic variation for this species is represented in the PE Carlos Botelho population [9], which was also identified as a demographic priority (see Table 3). Indeed, there is no comparable population of northern muriquis known to possess such a high level of that species’ genetic diversity. Other populations of southern muriquis identified for their genetic uniqueness, probably because of their histories, include São Sebastião, SP and Serra dos Orgãos, RJ, (Table 3). Additional populations of southern muriquis in each of the states of São Paulo, Rio de Janeiro, and Paraná may also emerge as priorities based on their suspected genetic uniqueness (e.g., Parque das Neblinas).

Considerations of geographic importance indicate Castro, in Paraná, as the southernmost population of *Brachyteles arachnoides* and of the genus. Similarly, southern muriquis in São Sebastião, SP (up to 2, 000 m above sea level) and Parque Nacional da Serra dos Órgãos, RJ, may include the highest altitudinal ranges. For southern muriquis, anchors for potential corridors include PECB, Castro, Fazenda Barreiro Rico, and PE Desengano (Table 3).

The feasibility of demographic monitoring of key southern muriqui populations is quite high in the state and national parks and at least one reserve. However, research access has been sporadic or restricted at some of the geographically important populations living in privately owned forests.

All else being equal, demographic monitoring programs will obviously be most successful for priority populations where implementability ratings are high (e.g., Legado das Águas Votorantim Reserve). Our assessment calls attention to the need for additional efforts to improve access, permissions and logistics in priority areas with low implementability (e.g., Fazenda Barreiro Rico) so that demographic monitoring programs for populations such as these can be initiated and maintained.

### Optimal monitoring intensity

Regardless of the frequency of observations or the duration over which population demography is monitored, the maintenance of consistent, detailed records is essential for analyses of demographic trends (See S1 Text). Interpretations of these demographic trends must be sensitive to differences in the intensity of monitoring and the accuracy of the demographic data obtained. At the most extreme monitoring intensity for muriquis (Table 4, far right column) are populations in which all individuals can be recognized by their natural markings and these individuals have been monitored for decades. In these cases, changes in group sizes and composition can be tracked on the basis of individual reproductive, survivorship, and dispersal events, often to the precision of days or weeks, depending on the monitoring frequency. However, in cases where such intensive monitoring may be unfeasible or undesirable, less intensive monitoring efforts can still yield valuable demographic data that permit systematic assessments of demographic trends (Table 4).

**Table 4:** Factors affecting the intensity of demographic monitoring and estimates of demographic trends.

Monitoring Intensity	Low←————————————————————————————————————→High				
Objective and Potential Analyses	Observed trends (increase or decrease) in population size	Observed trends *+ Predictions of population change (increase or decrease)*	Observed trends + Predictions *+ due to changes in fertility*	Observed trends + Predictions + *PVA*, *including age-specific mortality*	Observed trends + Predictions (fertility, age-specific mortality) *+ Individual life histories*
**Level of individual identification require**	None	Sex (for changes in sex ratios)	Sex + % females carrying infants	Sex + % females carrying infants + age-classes	Individual recognition
**Minimum frequency of successive monitoring campaigns**	Every 2–5 years	Annually	Annually	Annually	Ideally daily; up to monthly
**Minimum duration of monitoring necessary**	Corresponding to median interbirth interval (IBI); assumption is that populations may fluctuate with birth intervals (for muriquis, 3 yrs [28])	Corresponding to median interbirth interval (IBI); assumption is that populations may fluctuate with birth intervals (for muriquis, 3 yrs [28])	Corresponding to 2 IBIs (for muriquis, 6 yrs)	Corresponding to 2 IBIs (for muriquis, 6 yrs)	Corresponding to median female age at 1^st^ birth and median male age at 1^st^ complete copulation or youngest paternity (for muriquis, 9 years for female 5–8 years for male life histories [28,29])
**Other observations**	Monitor during the same season (ideally, during the same month) to control for effects of seasonality on births, deaths, and grouping patterns	Monitor during the same month each year	Monitor during the same month each year	Monitor during the same month each year	Monitor during the same days or weeks each month

The ability to recognize individuals may increase over time if the animals become habituated to observers and observers become more familiar with their subjects. However, poor visibility due to tall forest or dense vegetation, difficult terrain, or lack of distinctly visible markings can also preclude individual recognition. Southern muriquis, for example, have completely black faces and are therefore more difficult to identify individually than northern muriquis, which have distinct patterns of facial and genital depigmentation.

The advantages of being able to recognize individuals for demographic monitoring pertain mainly to precision, as the risks of over-estimating group and population size due to redundant counts of the same animals are reduced. Individual recognition also allows for the potential to monitor the variance in female reproductive rates, which can contribute to a better understanding of the processes, such as changes in fertility rates, that may underlie demographic trends [e.g., 2]. However, it is important to note that trends in population size can still be evaluated with counts of individuals in general, and projected trends in population size can be made based on changes in sex ratios, the percentage of fertile females (estimated from the percentage of females carrying infants), and calculations of age-sex class composition.

Trends in population sizes can be assessed whenever at least two counts of the population have been made, provided that these counts are conducted in a comparable, systematic ways. Thus, efforts to conduct the counts at the same time of day (due to diurnal variation in activities and its potential effects of visibility) or times of year (relative to seasonal variation in behavior and the seasonal timing of births) should be made to reduce potential sources of error in estimates of demographic trends.

Incorporation of muriqui life history data provides a rationale for interpreting population trends. For example, we can assume that if a population increases over the duration of a median IBI (i.e., 3 yrs for muriquis [28]), then it is likely that births (and immigrations) have outweighed deaths (and emigrations), whereas the opposite could be inferred if the population declined. Shorter monitoring durations would be less likely to detect these dynamics. Longer monitoring durations (minimally 6 years, corresponding to 2 IBIs for muriquis) are necessary to document changes in fertility patterns and even longer monitoring durations are necessary to document the effects of variation in individual life histories on demographic trends (Table 4).

## Recommendations and conclusions

One might suppose that, in an ideal world, with infinite access to trained personnel and financial and logistical resources, the most intensive monitoring schedule that can be implemented would always be preferred. However, local conditions should always be considered when deciding the frequency and intensity of demographic monitoring, paying particular attention to any factors that might make systematic monitoring undesirable or even potentially deleterious to the population, despite the importance of the documenting population trends. For example, populations living at extremely low density may not be good candidates for high intensity monitoring even if implementability scores are high. Other conditions might include, but are not limited to, ongoing pressures from hunters, which would make habituation difficult as well as ill-advised; similar risks of habituating the muriquis would also apply in populations where the animals are subjected to unregulated or poorly supervised visits from film crews, photographers, and eco-tourists. Thus, although there is no doubt that a population in which all individuals can be identified and monitored on a daily basis for decades will yield the most precise data on trends in the population’s size and composition, it is neither feasible nor optimal to strive to implement such intensive monitoring for all populations. Indeed, intensive monitoring should only be initiated in populations that are well protected and therefore not at increased risks from hunters as they become habituated to human observers.

The high level of habituation necessary for intensive monitoring of individuals requires confidence in the level of protection from hunters over the duration of the lifetimes of habituated individuals. However, even in well-protected populations, risks of over-habituation may increase vulnerability to infectious diseases or to physical harm from untrained observers (unsupervised tourists, for example). Thus, evaluations of the scientific and conservation “value added” from the initiation of new demographic monitoring programs (and from the continuation of ongoing programs) should be made on a regular basis [30].

Our criteria for assessing priority populations for demographic monitoring of the critically endangered northern and southern muriquis have been extremely useful in helping us to focus both new and ongoing research efforts. Our approach has also helped to reveal critical gaps in our knowledge of extant populations, such as those identified as being of high geographic importance but for which we lack demographic or genetic data (e.g., Reserva Biológica Augusto Ruschi and Fazenda Barreiro Rico for the northern and southern muriqui, respectively). Filling in these gaps may lead to shifts in which populations are prioritized, illustrating the dynamic nature of this process.

Although some criteria may differ, our approach is broadly applicable for other researchers engaged in the challenging process of prioritizing populations and of establishing criteria for integrated demographic monitoring programs of other species. We also show the relevance of considering life history data for each species to assess the most appropriate time scale at which the monitoring of populations and demographic trends can be most informative for conservation and management efforts.

## Supporting information

S1 Table Visible characteristics of muriqui developmental stages. (PDF)

S1 File Visible developmental stages in muriquis. Includes Figures A-R showing developmental stages for males and females.
(PDF)

S1 Text Maintaining records. (PDF)
