# Supplementary material for: Demographic monitoring of wild muriqui populations: Criteria for defining priority areas and monitoring intensity
Source: PLoS One. 2017 Dec 13;12(12):e0188922. doi: 10.1371/journal.pone.0188922 (PMC5728487; doi:10.1371/journal.pone.0188922)
Supplement: S1 Table — (PDF) [file pone.0188922.s001.pdf]

S1 Table: Visible Characteristics of Muriqui Developmental Stages.

Age category	Visible Characteristics
Infant	From 0-2 months, infants are carried ventrally by mothers and nursing frequently. By 6 months, infants are carried dorsally, riding jockey style; they may play and move next to mothers (Odália-Rimoli, 1998; Strier, 1987, 1992, 1993; Strier et al., 2006). They typically remain very near their mothers until 12 months, though they become increasingly independent in feeding and traveling. Infants continue to nurse and to be carried jockey style on their mothers' back until weaning, which occurs between 12 and 24 months (Odália-Rimoli, 1998; Strier et al., 2006). Mothers may continue to carry and help infant in difficult tree crossing after weaning (Strier, 1987). Odália-Rimoli (1998) notes that the second year of life is marked by less contact with mother.
Juvenile	Juveniles by 3 years are fully weaned and independent (Strier, 1992). They have fully independent locomotion (Strier 1987),but may continue to travel near their mothers until a new sibling is born (Odália-Rímolí, 1998; Strier et al. 2006). Following Strier (1987), young juveniles (approximately 2- 3 ½ years) and older juveniles/adolescents (3 ½ -6 years) are mainly distinguished by their body size. Between 3-4 years of age the first signs of facial depigmentation are evident, but only for northern muriquis. Southern muriquis retain their black faces for much longer, with occasional depigmentation on genital regions (Talebi et al., 2005). Juveniles are sexually immature (Tolentino et al., 2008).
Subadult	Smaller than fully grown adults, females have small but visible nipples and the genitalia is smaller and less pendulous than adults, but resembles that of adults in proportions (Strier, 1986, 1993). Young immigrant subadult females engage frequently in play groups and their body size is slightly smaller than adults. Females at RPPN-FMA transfer at an average age of 6.12 years (Strier et al., 2006), prior to the onset of puberty (Strier and Ziegler, 2000). Strier (1987) noted that the testes of subadult males are larger than those of juveniles but smaller than those of fully mature adult males. Males as young as 5 years may already be completing copulations (Strier and Mendes, 2012), but the youngest known sire in this population was just over 8 years old (Strier, et al., 2011).
Adult	Clitoris and labia of females are elongated and males testes are equivalent to softballs when extended (Strier, 1986; 1987). At RPPN-FMA, we have used age at first complete copulation for males (Possamai, et al. 2005; Tolentino, et al., 2008) and first copulation (known to coincide with the onset of cycling) for females to define the onset of adulthood, although median age at first reproduction for females is roughly 9 years (Strier et al. 2006; Strier and Mendes, 2012).

Visible, physical characteristics are probably the most reliable method for classifying muriquis by age-sex class (See below, and Figures A-R in S1 File). As a result of long-term studies of recognized individual muriquis in the Matão group at the RPPN-FMA since 1983, we also now have data on a number of behavioral milestones, such as the age at which males begin to complete copulations (copulate with ejaculation). Clearly, such specific behavioral milestones are of limited use for distinguishing adults from subadult males in other groups or populations without comparable close observations. However, other behavioral milestones, such as when infants typically move from being carried ventrally to being carried dorsally, are more feasible to observe regardless of monitoring

intensity. Thus, we include such behavioral as well as physical characteristics in our descriptions of age-sex classes.

We expect these descriptions will evolve over time, as more data from other populations become available. Indeed, we have only begun to understand the life histories of muriquis, and to pair their behavioral, hormonal, and reproductive milestones with visible physical developmental features. We know that male muriquis in captivity can exhibit more precocial sexual maturation than we have seen in the wild (Pissinati, et al., 1998), but we still do not know whether developmental stages in muriquis in the RPPN-FMA population are representative of those in other populations of wild northern and southern muriquis.

References:

Odália-Rimoli A. Desenvolvimento Comportamental do Muriqui (Brachyteles arachnoides) na Estação Biológica de Caratinga, Minas Gerais. Tese de Doutorado, Universidade de São Paulo, 1998.

Pissinatti A, Coimbra-Filho AF, Rylands AB. Observations on reproduction and behavior of the muriqui, *Brachyteles arachnoides*, in captivity. *Neotropical Primates* 1998; 6: 40-45.

Possamai CB, de Oliveira RCR, Mendes SL, Strier KB, Young RJ. Age-related variation in copulations of male northern muriquis (*Brachyteles hypoxanthus*). Folia Primatol 2005; 76: 33-36.

Strier KB. Demographic patterns of one group of free-ranging woolly spider monkeys. Primate Conservation 1987; 8:73-84.

Strier KB. Faces in the Forest: The endangered muriqui monkey of Brazil. New York Oxford University Press, 1992.

Strier KB. Growing up in a patrifocal society: sex differences in the spatial relations of immature muriquis (*Brachyteles arachnoides*). In Pereira ME, Fairbanks LA, editors. Juveniles primates: Life history, development and behavior, New York, Oxford University Press, 1993, pp. 138-147.

Strier KB, Mendes S.L. The northern muriqui (*Brachyteles hypoxanthus*): Lessons on behavioral plasticity and population dynamics from a critically endangered primate. In Kappeler P, Watts D, editors. Long-term studies of primates. Heidelberg, Springer-Verlag, 2012, pp. 125-140.

Strier KB, Ziegler TE. Lack of pubertal influences on female dispersal in muriqui monkeys (*Brachyteles arachnoides*). Anim Behav, 2000; 59:849-860.

Strier KB, Boubli JP, Possamai CB. Mendes SL. Population demography of northern muriquis (*Brachyteles hypoxanthus*) at the Estação Biológica de Caratinga/Reserva Particular do Patrimônio Natural- Feliciano Miguel Abdala, Minas Gerais, Brasil. Am J Phys Anthro 2006;130:227-237.

Strier KB, Chaves PB, Mendes SL, Fagundes V, Di Fiore A. Low paternity skew and the influence of maternal kin in an egalitarian, patrilocal primate. Proc Nat Acad Sci USA, 2011; 108:18915-18919.

Talebi M, Bastos A, Lee PC. Diet of southern muriquis in continuous Brazilian Atlantic forest. Int J Primatol 2005 ;26(5): 1175-1187.

Tolentino K, Roper JJ, Passos FC, Strier KB. Mother-offspring associations in northern muriquis, *Brachyteles hypoxanthus*. Am J Primatol 2008; 70:301-305.
