# Supplementary material for: Demographic monitoring of wild muriqui populations: Criteria for defining priority areas and monitoring intensity
Source: PLoS One. 2017 Dec 13;12(12):e0188922. doi: 10.1371/journal.pone.0188922 (PMC5728487; doi:10.1371/journal.pone.0188922)
Supplement: S1 File — Includes Figures A-R showing developmental stages for males and females. (PDF) [file pone.0188922.s002.pdf]

S1 File: Visible Developmental Stages in Muriquis

The use of high resolution photographic images and of artistic renditions in the identification of visible developmental features is still at an early stage for muriquis. Nonetheless, these tools are vital to insure that the data obtained from our integrated demographic monitoring program for muriquis is reliable over time and across populations. Researchers interested in initiating a population monitoring program at any of the areas identified as priorities are encouraged to gain some experience in observing individuals of known ages, either at RPPN-FMA or one of the other ongoing study sites, or at CPRJ. This will facilitate consistency in the assignment of age-sex classes across populations, and increase the accuracy of the integrated demographic monitoring program overall (see S1 Table, and Figures A-R in S1 File).

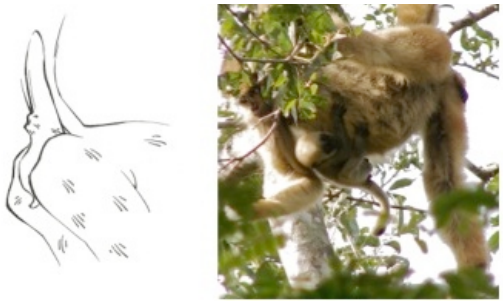

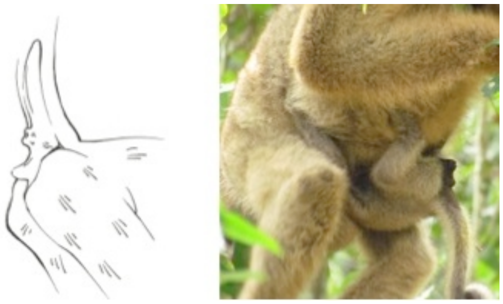

Figures A and B. Genitalia of northern muriquis from RPPN-FMA at < 1 month of age. Male is shown on left (Figure A); female on right (Figure B). Illustrations show the different positions and shape of the male and female genitalia at this age, when infants are carried almost exclusively ventrally (see S1 Table). Photos by Carla B. Possamai; drawings by André Ferreira.

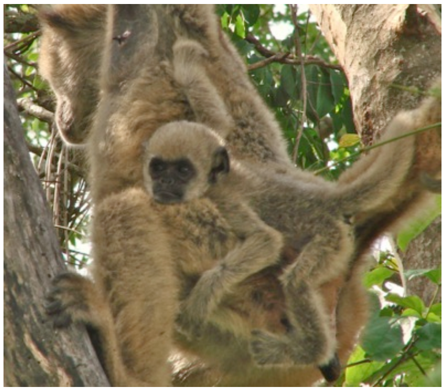

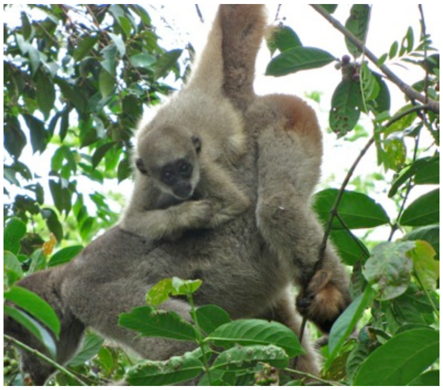

Figures C and D. Northern muriquis at RPPN-FMA at 6 months of age. Male is shown on left (Figure C); female on right (Figure D). By this age, infants are usually carried on their mothers' backs with their tails wrapped around their mothers' tails during locomotion, but they also leave contact and explore on their own when their mothers are resting (see S1 Table). Photos by Carla B. Possamai.

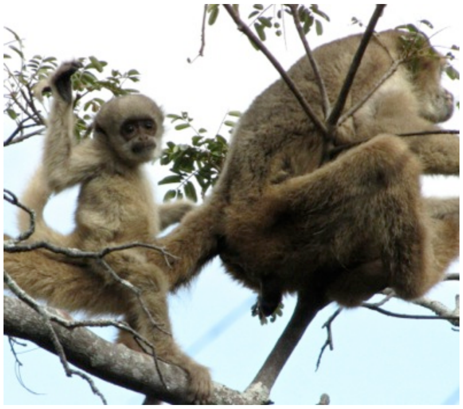

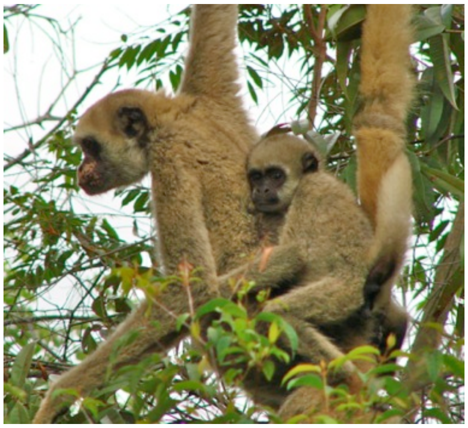

Figures E and F. Northern muriquis at RPPN-FMA at 1 year of age.   Male is shown on left (Figure E); female on right (Figure F). By this age, individuals are increasingly independent; they explore their environment and increasingly eat on their own. See references in S1 Table. Photos by Carla B. Possamai.

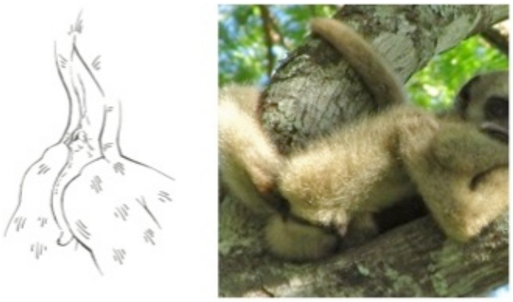

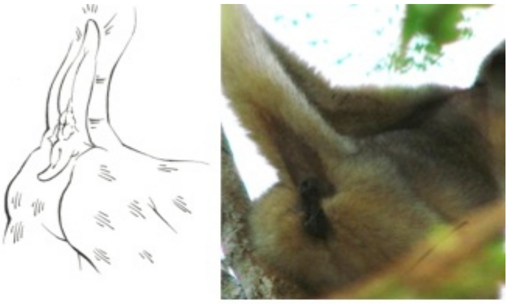

Figures G and H. Genitalia of northern muriquis from RPPN-FMA at 2 years of age. Male is shown on left (Figure G); female on right (Figure H). By this age, infants are increasingly independent and most have undergone, or are in the process of undergoing weaning (see S1 Table). Photos by Carla B. Possamai; drawings by André Ferreira.

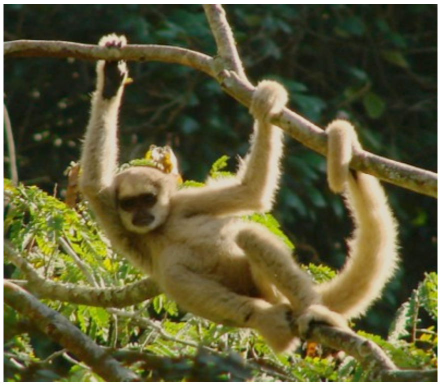 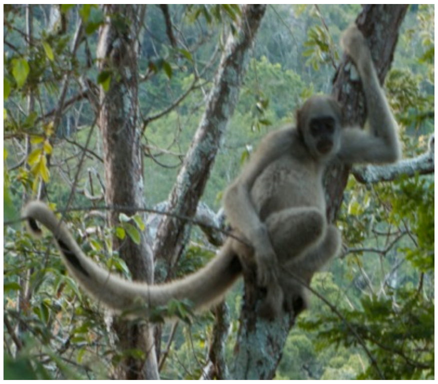

Figures I and J. Northern muriquis at RPPN-FMA at 3 years of age. Male is shown on left (Figure I); female on right (Figure J). By this age, muriquis are fully independent; they feed and travel separate from their mothers. It is also possible to see the beginnings of their facial dispigmentation (S1 Table). Photos by Carla B. Possamai.

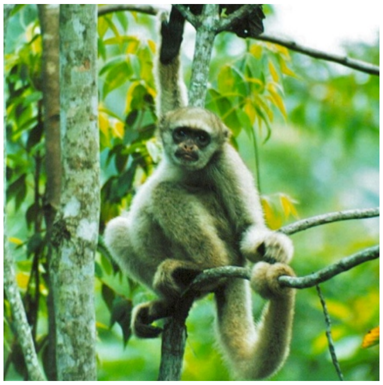 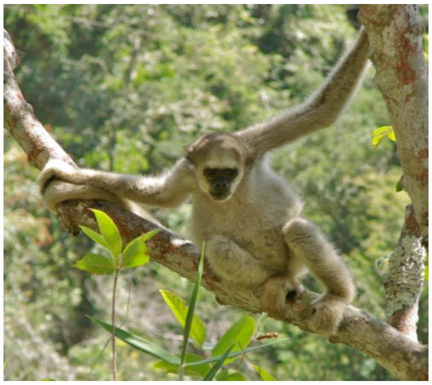

Figures K and L. Northern muriquis at RPPN-FMA at 4 years of age. Male is shown on left (Figure K); female on right (Figure L). By this age, muriquis are fully independent and their faces show the beginnings of dispigmentation (S1 Table). Photos by Carla B. Possamai.

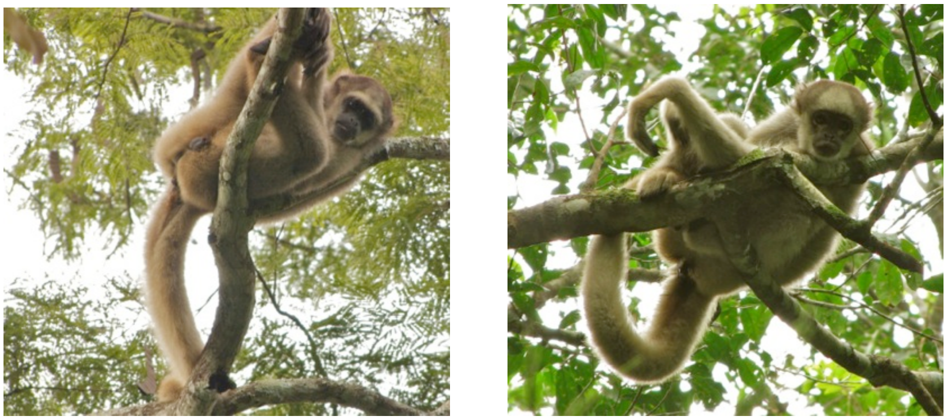

Figures M and N. Northern muriquis at RPPN-FMA at 5 years of age. Male is shown on left; (Figure M) female on right (Figure N). At this age, their bodies are still smaller than those of adults but their genitalia have begun to increase in size and general maturation is visible (S1 Table). Photos by Carla B. Possamai.

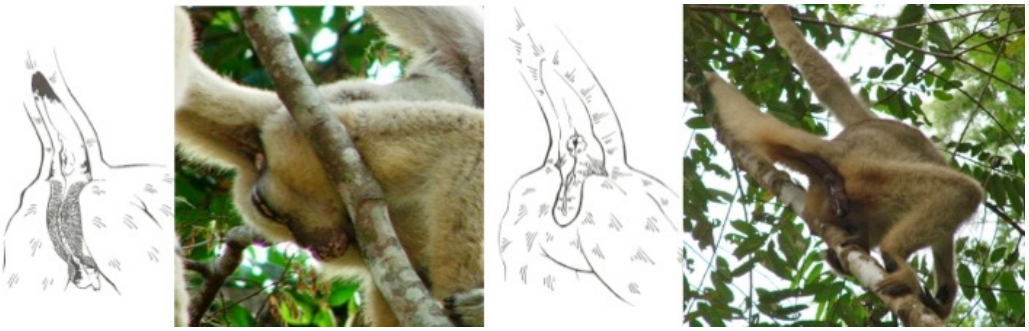

Figures O and P. Genitalia of northern muriquis from RPPN-FMA at 6 years of age. Male is shown on left (Figure O); female on right (Figure P). At this age, the genitals of both males and females are close to adult size and shape; female nipples are also more evident than in younger individuals (see S1 Table). Photos by Carla B. Possamai; drawings by André Ferreira.

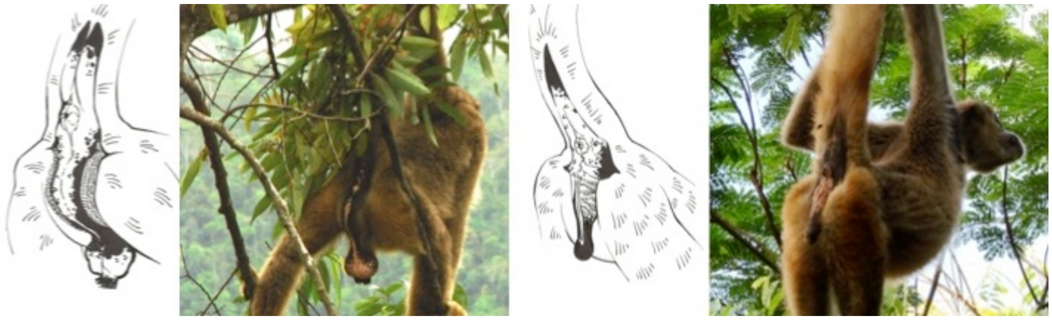

Figures Q and R. Genitalia of northern muriquis from RPPN-FMA at > 6 years of age. Male is shown on left (Figure Q); female on right (Figure R). Testicles are completely developed in males and the clitoris is elongated in females; the nipples of females that have nursed infants are also elongated (see S1 Table). Photos by Carla B. Possamai; drawings by André Ferreira.
