# Supplementary material for: Demographic monitoring of wild muriqui populations: Criteria for defining priority areas and monitoring intensity
Source: PLoS One. 2017 Dec 13;12(12):e0188922. doi: 10.1371/journal.pone.0188922 (PMC5728487; doi:10.1371/journal.pone.0188922)
Supplement: S1 Text — (PDF) [file pone.0188922.s003.pdf]

S1 Text: Maintaining Records

When demographic data are limited to counts of individuals or of individuals that can be distinguished by age-sex class, the kind of "ficha" described in Tabacow, et al. (forthcoming) is suitable. When demographic data can be collected on recognized individuals, and individual-based "biography" table, as described in Strier, et al. (2010) may be ideal (see example at http://demo.plhdb.org).

The main distinction between these two approaches to maintaining demographic records is that in the first case, each line (record) is a group or population count, where in the second case, each line (record) is an individual. Summing all individuals alive at any time will yield the group or population size; separating individuals by age (calculated from known or estimate birthdates) will yield data on the size of different age-sex classes present at any time. "Biography" tables lose some of their utility when there are big differences between minimum and maximum estimated birthdates, and thus, may not be well-suited when populations (or groups) are monitored at widely spaced intervals or when the probability of missing a birth record (because the infant was born and died between observation intervals) is high.

References:

Strier KB, Altmann J, Brockman DK, Bronikowski A, Cords M, Fedigan LM, Lapp H, Liu X, Morris WF, Pusey AE, Stoinski TS, Alberts SC. The primate life history aatabase: A unique shared ecological data resource. *Methods Ecol Evol* 2010: 199-210.

Tabacow FP, Melo FR, Moreira LS, Strier KB, Possamai CB, Breves P, Jerusalinsky, L. Protocolo padronizado para contagem de indivíduos de *Brachyteles* spp. Ação 1.4 do Plano de Ação Nacional para a Conservação dos Muriquis, ICMBio (forthcoming).
